# Comparison of sodium removal in peritoneal dialysis patients treated by continuous ambulatory and automated peritoneal dialysis

**DOI:** 10.1007/s40620-019-00646-7

**Published:** 2019-09-09

**Authors:** Sarju Raj Singh Maharjan, Andrew Davenport

**Affiliations:** UCL Department of Nephrology, Royal Free Hospital, University College London, Rowland Hill Street, London, NW3 2PF UK

**Keywords:** Peritoneal dialysis, Sodium, Ultrafiltration, Blood pressure, CAPD, APD, Icodextrin, Hypertonic glucose

## Abstract

**Background:**

Optimal fluid balance for peritoneal dialysis (PD) patients requires both water and sodium removal. Previous studies have variously reported that continuous ambulatory peritoneal dialysis (CAPD) removes more or equivalent amounts of sodium than automated PD (APD) cyclers. We therefore wished to determine peritoneal dialysate losses with different PD treatments.

**Methods:**

Peritoneal and urinary sodium losses were measured in 24-h collections of urine and PD effluent in patients attending for their first assessment of peritoneal membrane function. We adjusted fluid and sodium losses for CAPD patients for the flush before fill technique.

**Results:**

We reviewed the results from 659 patients, mean age 57 ± 16 years, 56.3% male, 38.9% diabetic, 24.0% treated by CAPD, 22.5% by APD and 53.5% APD with a day-time exchange, with icodextrin prescribed to 72.8% and 22.7 g/L glucose to 31.7%. Ultrafiltration was greatest for CAPD 650 (300–1100) vs 337 (103–598) APD p < 0.001, vs 474 (171–830) mL/day for APD with a day exchange. CAPD removed most sodium 79 (33–132) vs 23 (− 2 to 51) APD p < 0.001, and 51 (9–91) for APD with a day exchange, and after adjustment for the CAPD flush before fill 57 (20–113), p < 0.001 vs APD. APD patients with a day exchanged used more hypertonic glucose dialysates [0 (0–5) vs CAPD 0 (0–1) L], p < 0.001.

**Conclusion:**

CAPD provides greater ultrafiltration and sodium removal than APD cyclers, even after adjusting for the flush-before fill, despite greater hypertonic usage by APD cyclers. Ultrafiltration volume and sodium removal were similar between CAPD and APD with a day fill.

## Introduction

Peritoneal dialysis (PD) is an established treatment for patients with end-stage kidney disease. In addition to obtaining adequate solute clearances, PD should control volume status and sodium balance. After peritonitis [1], failure to achieve adequate ultrafiltration is the next commonest cause of PD technique failure, and just as there are targets for small solute clearances for PD patients the European Automated Peritoneal Dialysis Outcomes Study (EAPOS) recommended a minimum target amount of ultrafiltration to prevent volume overload and PD technique failure [2].

Patients with faster peritoneal transport are reported to have lower technique survival when treated by continuous ambulatory peritoneal dialysis (CAPD) [3]. Longer cycle dwell times risk a reduction in the osmotic gradient that drives ultrafiltration and sodium removal in faster peritoneal transporters treated by CAPD, due to glucose absorption. Failure to achieve adequate sodium removal will lead to extracellular water (ECW) expansion and hypertension, resulting in left ventricular hypertrophy [4] with increased risk of cardiovascular and cerebrovascular events in dialysis patients [5]. The introduction of automated peritoneal dialysis (APD) cyclers has been reported to reduce technique failure rates for faster peritoneal transporters [3], and studies from both the USA and Brazil have reported greater PD technique and patient survival with APD compared to CAPD [6, 7], even after adjustment for patient demographics and co-morbidity [8].

There have been a limited number of studies which have specifically addressed sodium removal by different modes of PD; It has been suggested that CAPD is more effective than APD in terms of both ultrafiltration volumes and sodium removal, probably due to the longer cycle dwell times [9–11]. Although these studies may have over-estimated the sodium removal by CAPD by not taking into account the volume used for the flush before fill technique used with CAPD exchanges. Other studies have observed no difference in sodium removal and volume status comparing CAPD and APD with a day time exchange [12]. The differences between studies may reflect the use of 7.5% icodextrin, which has been reported to increase peritoneal sodium removal compared to glucose dialysates for both CAPD and APD patients with a day time exchange [13].

We therefore wished to determine whether sodium removal differed between the different PD modalities, and whether there was any association between sodium removal and ECW excess or hypertension.

## Materials and methods

We retrospectively audited consecutive PD outpatients treated with CAPD, APD and APD with a day time exchange (continuous cycling peritoneal dialysis—CCPD) attending for their first assessment of peritoneal membrane function. All patients had a 22.7 g/L exchange prior to attendance. No patient had suffered with peritonitis in the previous 8 weeks or an emergency admission to hospital. We excluded patients with implantable cardiac devices, amputations and those unable to stand.

Patients had standing height measured and were weighed post voiding and with peritoneal dialysate drained out. PD adequacy was calculated by standard methods from 24-h urinary collections and samples from spent dialysates and estimated normalised protein nitrogen appearance (nPNA) [14]. Peritoneal membrane transport was calculated from 4-h peritoneal dialysate dwell and plasma creatine concentrations using a standard 2.0 L 22.7 g/L peritoneal dialysate [14]. In addition to standard biochemical tests, we also measured blood glucose, serum albumin by bromocresol green method and creatinine enzymatically (Roche Modular P^®^ analyser, Roche Diagnostics Limited, Burgess Hill, UK), with sodium in urine and dialysates measured using an indirect ion electrode [15]. Serum sodium values were adjusted if serum glucose was elevated [16]. Sodium removal was calculated by the addition of 24-h urinary sodium to the difference between dialysate sodium instilled and the sodium in 24-h effluent dialysate. Patients and staff were instructed to allow 15 s for the flush before fill CAPD technique, and the median volume measured was 90 mL, as such sodium balance in CAPD patients was then adjusted from an initial volume of 2.15 L in a fresh dialysate bag [17]. We calculated the amount of glucose in the dialysis prescription and report this as glucose exposure (mmol/day). No patient was prescribed a glucose dialysate concentration above 22.7 g/L.

Multifrequency bioelectrical impedance (MFBIA) was measured using a standardised protocol (InBody 720, Seoul, South Korea), with dialysate drained out and after voiding [18, 19]. Blood pressure was recorded in the supine position after the patient had drained out dialysate and rested for a minimum of 30 min and abstained from any stimulants (Dinamap, Critikon Corporation, Tampa, FL, USA). All equipment was regularly serviced and calibrated.

Medications were obtained from hospital computerised records. All patients were provided with dietary advice, from a renally trained dietician, to limit dietary sodium to around 100 mmol/day, and loop diuretics (250 mg/day frusemide) were prescribed as standard treatment for patients with urinary output of ≥ 200 mL/day.

### Statistical analysis

Results are expressed as mean ± standard deviation, or median and interquartile range, or percentage. We used standard statistical analysis D’Agostino and Pearson normality test, Chi square analysis with adjustment for small numbers where appropriate. Anova and Kruskal–Wallis analyses for parametric and nonparametric group data, with appropriate correction for multiple analyses by Tukey or Games–Howell ad hoc testing respectively. Univariate analysis was performed by Spearman’s correlation for adjusted PD sodium losses and followed by a multivariable step-backward regression model for higher than median vs lower than median adjusted peritoneal dialysate sodium losses. Variables with univariate association of p < 0.1 were included. Non-parametric data was log transformed, and variables excluded in a back-ward regression model if not significant and did not improve model fit. The model was checked for collinearity and variance inflation factor. Statistical analysis was performed using Graph Pad Prism (version 8.1, Graph Pad, San Diego, CA, USA) and Statistical Package for Social Science version 24.0 (IBM Corporation, Armonk, NY, USA). Statistical significance was taken at or below the 5% level.

### Ethics

Our retrospective audit of service development complied with the United Kingdom National Health Service Health Research Authority, guidelines for clinical audit and service development. with all patient data anonymised prior to analysis (https://www.hra.nhs.uk), and complied with United Kingdom National Institute for Clinical Excellence best practices, http://www.nice.org.uk/media/796/23/bestpracticeclinicalaudit.pdf, and registered with the University College department of nephrology.

## Results

**Fig. 1:**
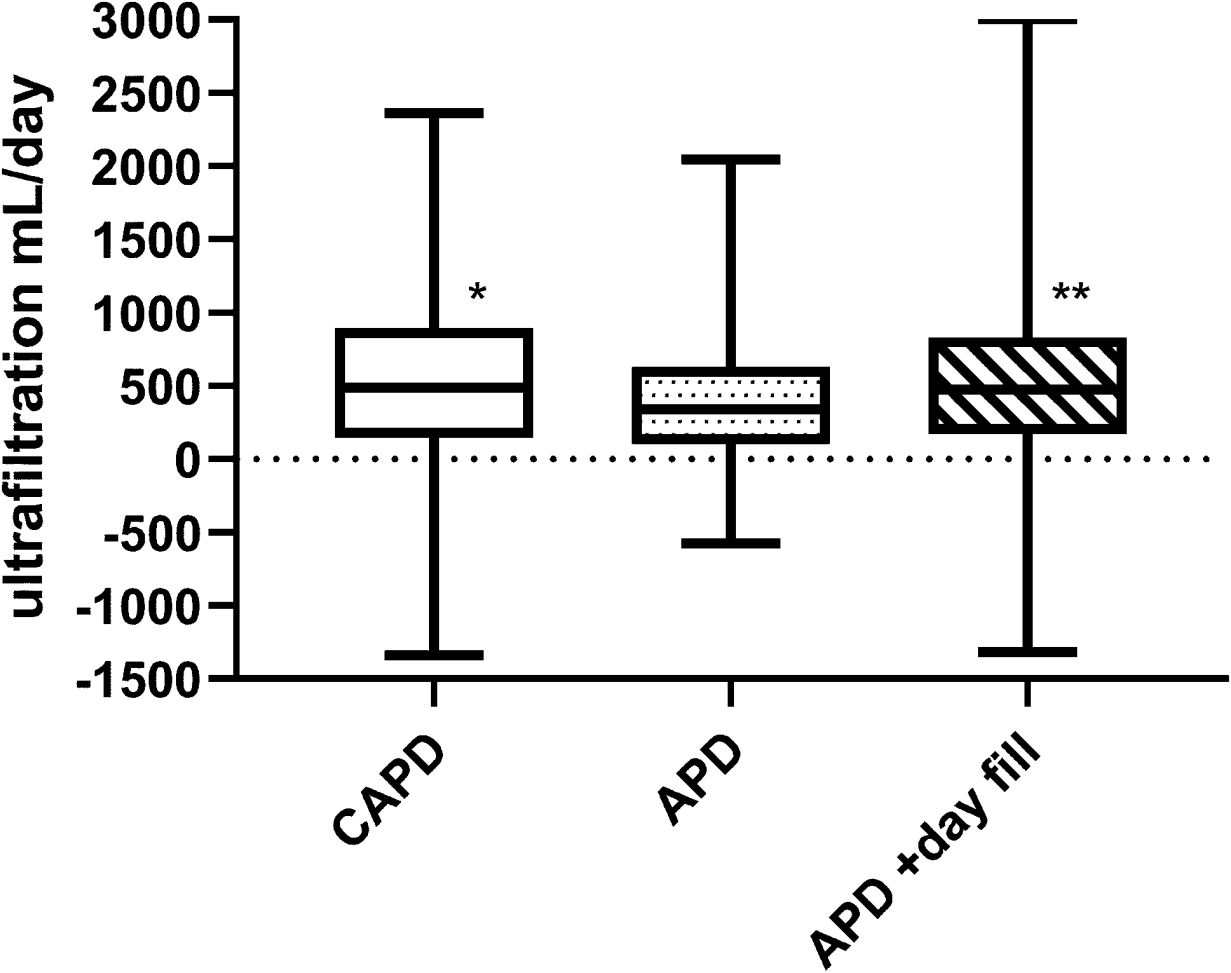
Twenty-4-h peritoneal dialysate ultrafiltration volumes. Continuous ambulatory peritoneal dialysis (CAPD), automated peritoneal dialysis (APD) cyclers. Adjusted CAPD accounts for the flush before fill technique., **p < 0.01 vs APD with a day time exchange

**Fig. 2:**
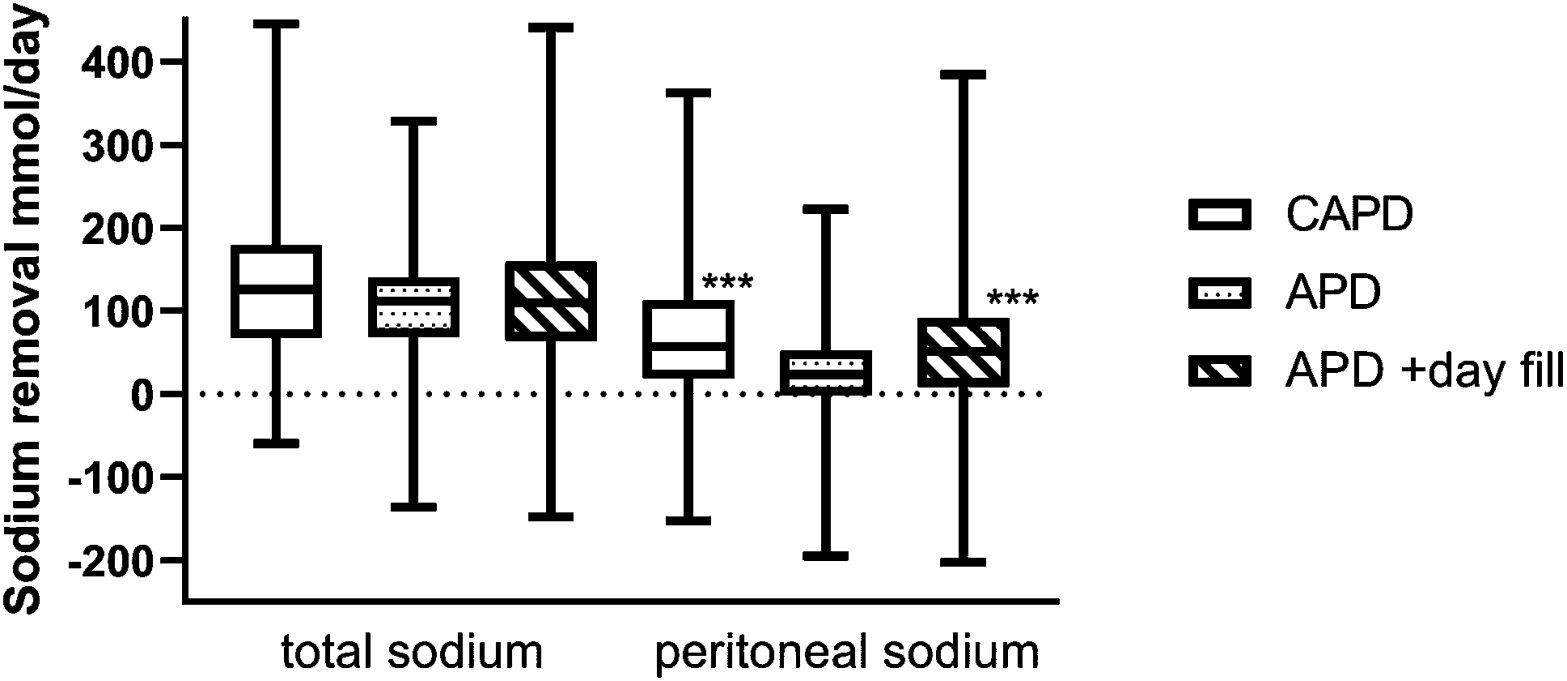
Twenty-4-h sodium balance as the difference between sodium losses in urine and peritoneal dialysate minus sodium infused in peritoneal dialysate, and 24-h peritoneal sodium balance as the difference between sodium in drained peritoneal dialysate minus sodium infused in peritoneal dialysate. Continuous ambulatory peritoneal dialysis (CAPD), automated peritoneal dialysis (APD) cyclers. Adjusted CAPD accounts for the flush before fill technique., ***p < 0.001 vs APD with a day time exchange

We reviewed the data on 659 adult PD patients attending for their first assessment of peritoneal membrane function, median duration of peritoneal dialysis 3 (2–7) months. The majority of patients were treated by APD with a day-time exchange (Table 1). Patients dialysed daily, and the median APD cycler session time was 8.0 (8.0–8.0) h. Patients treated by CAPD were older. The majority of CAPD patients and those treated by APD with a day-time exchange were prescribed icodextrin, and more hypertonic glucose exchanges were used by APD patients with a day-time exchange. Patients treated by APD had greater urine output and urinary sodium excretion. Net peritoneal ultrafiltration and sodium removal was greatest with CAPD, although this then fell after adjustment for the fill before flush technique, net ultrafiltration volume and peritoneal sodium loss remained greater for CAPD compared to APD (p < 0.001), but not for APD with a day-time icodextrin exchange (Figs. 1, 2). Peritoneal transporter status, the number of diabetic subjects, body composition and nPNA did not differ between the different PD treatment modes of treatment. Total weekly Kt/V_urea_ was similar between the different PD modes (Table 1), as was daily net sodium removal, after adjustment for the flush before fill (Fig. 2).

**Table 1:** Patient demographics, blood pressure, residual renal function, peritoneal clearance and transport status

Variable	All	CAPD	APD	CCPD
Number	659	158	148	353
Male (%)	371 (56.3)	83 (52.5)	79 (53.4)	207 (58.6)
Age (years)	57 ± 16	62 ± 17***	58 ± 16**	54 ± 15
Diabetic (%)	253 (38.9)	66 (42.0)	43 (27.6)*	134 (38.0)
Icodextrin (%)	480 (72.8.2)	146 (91.8)^+++^	0***	334 (94.4)
22.7 g/L glucose (%)	209 (31.7)	40 (25.2.2)*	23 (15.5)*	146 (41.2)
Cycles/exchanges	7 (5–7)	4 (2–4)***	6 (6–7)	7 (6–8)
Cycle/exchange volume (L)	2 (2–2)	2 (2–2)	2 (2–2)	2 (2–2)
Icodextrin (L/day)	1.2 (0–2)	2 (2–2)^+++^	0 (0–0)	2 (2–2)
22.7 g/L glucose (L/day)	0 (0–4)	0 (0–1)***	0 (0–0)***	0 (0–5)
13.6 g/L glucose (L/day)	7.2 (4.0–9.3)	3 (0–6)***	9 (6–10)	8 (5–10)
Glucose exposure (mmol/day)	690 (531–905)	300 (0–450)***	705 (600–796)	813 (675–1050)
Urine (mL/day)	1069 (530–1628)	1149 (591–1655)*	1172 (758–1722)***	911 (409–1498)
Urine sodium mmol/day	55 (25–99)	50 (22–96)^++^	78 (43–112)***	47 (19–96)
Weekly (Kt/Vurine)	1.18 (0.64–1.89)	1.3 (0.73–1.9)*	1.52 (0.94–2.12)***	1.01 (0.43–1.71)
Weekly (Kt/V_PD_)	1.18 (0.91–1.47)	1.10 (0.59–1.5)***	1.02 (0.83–1.23)***	1.28 (1.05–1.58)
Total weekly (Kt/V)	2.38 (1.91–3.07)	2.37 (1.93–3.05)	2.53 (1.94–3.14)	2.34 (1.91–3.02)
nPNA (g/kg/day)	0.92 ± 0.25	0.95 ± 0.25	0.96 ± 0.25**	0.89 ± 0.24
D4/P_creatinine_	0.73 ± 0.19	0.79 ± 0.29	0.62 ± 0.13***	0.75 ± 0.12
BMI (kg/m^2^)	25.8 ± 4.0	25.6 ± 4.3	25.8 ± 4.8	27.5 ± 5.5
MAP (mmHg)	101 ± 17	99 ± 17	100 ± 15	102 ± 17
SMMI (kg/m^2^)	9.6 ± 1.3	9.4 ± 1.5	9.6 ± 1.6	9.8 ± 1.7
FMI (kg/m^2^)	8.2 ± 4.1	9.4 ± 1.5	8.0 ± 3.9	9.8 ± 4.1
Albumin (g/L)	38 (35–41)	38 (34–41)	39 (36–42.5)***	37 (34–40)
CRP (mg/L)	1 (4–9)	5 (2–11)	2 (1–8)	3 (1–8)
Haemoglobin (g/L)	112.0 ± 15.2	112.9 ± 13.7	113.4 ± 15.9	111.0 ± 16.8

Continuous ambulatory peritoneal dialysis (CAPD) automated peritoneal dialysis (APD) and APD with a day-time exchange (CCPD). Weekly urea clearance (Kt/V), 4 h peritoneal equilibrium test dialysate/plasma creatinine ratio (4 h D/P_creatinine_), normalised nitrogen appearance rate (nPNA), body mass index (BMI), mean arterial blood pressure (MAP), skeletal muscle mass index (SMMI), fat mass index (FMI) and C reactive protein (CRP)

*p < 0.05, **p < 0.01, ***p < 0.001 vs CCPD

^+^p < 0.05, ^++^p < 0.01, ^+++^p < 0.001 vs APD

Dividing patients according to peritoneal transporter status [14], then more faster transporters were male and diabetic, and treated by CAPD and APD with a day time exchange, use of icodextrin and hypertonic glucose exchanges, but there were no over-all differences in sodium removal (Table 2). Sub-dividing patients according to transporter status and PD treatment mode, there were very few slow transporters, and as such we combined slow and slow-average transporters. There were no differences between peritoneal sodium losses between the different PD treatment modes for slow and slow-average transporters, but peritoneal sodium removal was lower for fast-average and fast transporters for those treated with APD cyclers (Table 2).

**Table 2:** Patients grouped according to peritoneal transport status [14]

Variable	Slow	Slow-average	Fast-average	Fast
Number	35	165	285	174
Male (%)	9 (25.7)***	86 (52.1)	160 (56.1)	114 (65.5)
Age (years)	53 ± 17	54 ± 17*	57 ± 15	60 ± 17
Diabetic (%)	8 (22.9)**	48 (29.1)**	113 (39.6)	76 (43.7)
CAPD/APD/CCPD (%)	9/71/20***	17/44/40	24/13/63	35/8/58
Cycles/exchanges	6 (5–7)	6 (5–7)	7 (5–7)	6 (5–7))
Cycle volume (L)	2 (2–2)	2 (2–2)	2 (2–2)	2 (2–2)
Icodextrin (L/day)	0 (0–0)***	0 (0–2)***	0 (1–2)*	2 (1–2)
22.7 g/L glucose (L/day)	0 (0–0)	0 (0–0)	0 (0–4)	0 (0–4)
13.6 g/L glucose (L/day)	9 (6–10)**	8 (5–9)*	7 (4–9)	5 (4–6)
Glucose exposure (mmol/day)	686 (540–844)	675 (563–844)	714 (552–965)	686 (402–900)
Urine (mL/day)	1069 (831–1500)	1087 (623–1677)	1060 (493–1657)	917 (410–1496)
Urine sodium (mmol/day)	53 (43–98)	61 (32–98)	54 (21–102)	46 (19–85)
Weekly (Kt/Vurine)	1.59 (0.83–1.89)	1.3 (0.73–1.9)*	1.18 (0.96–1.83)	0.97 (0.47–1.67)
Weekly (Kt/V_PD_)	1.1 (0.89–1.3)**	1.08 (0.81–1.3)**	1.21 (0.96–1.48)	1.33 (0.98–1.64)
Total weekly (Kt/V)	2.69 (2.1–3.4)	2.42 (1.94–3.14)	2.34 (1.93–3.03)	2.36 (1.83–3.0)
nPNA (g/kg/day)	0.92 ± 0.28	0.94 ± 0.27	0.90 ± 0.23	0.89 ± 0.25
Peritoneal sodium removal (mmol/day)	44 (17–76)	35 (10–75)*	42 (2–86)	52 (18–104)
Total sodium removal (mmol/day)	117 (78–142)	112 (73–151)	112 (61–168)	113 (66–160)
BMI (kg/m^2^)	25.1 ± 4.1	26.7 ± 5.1	26.3 ± 4.8	25.5 ± 4.6
MAP (mmHg)	101 ± 17	99 ± 17	100 ± 15	102 ± 17
SMMI (kg/m^2^)	9.1 ± 1.4	9.8 ± 2.5	9.7 ± 2.6	9.8 ± 1.5
FMI (kg/m^2^)	8.2 ± 3.6	8.9 ± 4.1***	8.4 ± 4.1**	7.1 ± 3.8
Albumin (g/L)	41 (39–44)***	40 (36–42)***	37 (35–40)	35 (32–39)
CRP (mg/L)	2 (1–6)	4 (1–8)	4 (1–9)	4 (2–10)
Haemoglobin (g/L)	116.0 ± 17.5	115.5 ± 15.9	110.7 ± 16.2	110.0 ± 15.2

Peritoneal sodium removal adjusted for flush before fill technique. Continuous ambulatory peritoneal dialysis (CAPD) automated peritoneal dialysis (APD) and APD with a day-time exchange (CCPD). Weekly urea clearance (Kt/V), normalised nitrogen appearance rate (nPNA), body mass index (BMI), mean arterial blood pressure (MAP), skeletal muscle mass index (SMMI), fat mass index (FMI) and C reactive protein (CRP)

*p < 0.05, **p < 0.01, ***p < 0.001 vs fast transporters

**Fig. 3:**
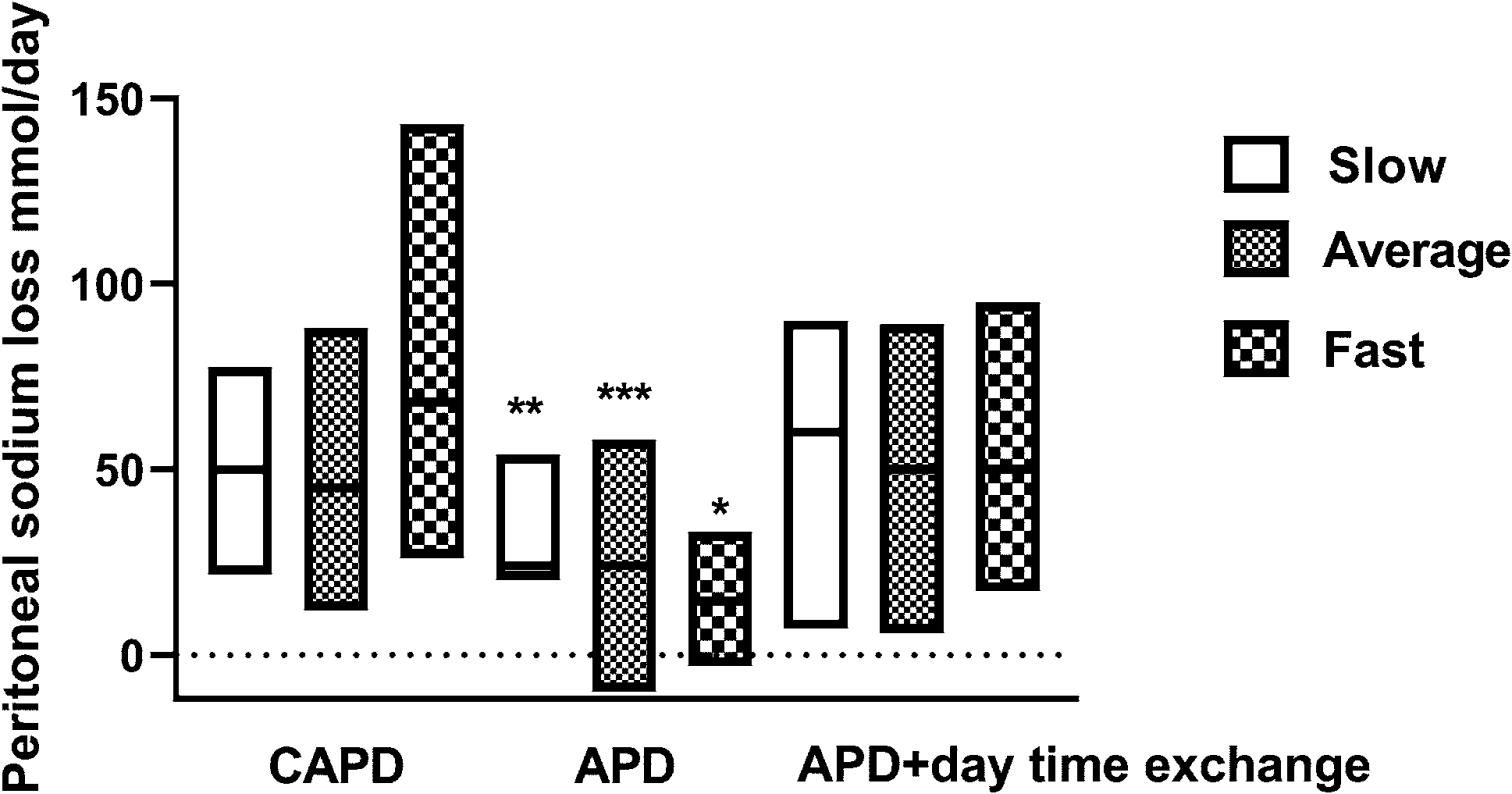
The effect of peritoneal dialysis modality and peritoneal dialysis transporter status using European Best Practice Guideline definition of slow, average and fast transporter [20]. Continuous ambulatory peritoneal dialysis (CAPD), automated peritoneal dialysis (APD). *p < 0.05, **< 0.01, < 0.001 vs CAPD fast transporter

To determine the effect of peritoneal transport status and PD modality we used the European Dialysis and Transplant best practice guideline definitions of slow, average and fast transporter [20]. Peritoneal sodium losses were greater for faster CAPD transporters compared to APD (Fig. 3), and slow CAPD transporters were prescribed less glucose compared to patients treated by APD with and without a day time exchange (Slow CAPD vs slow APD p < 0.05, vs average APD and slow APD with day exchange p < 0.01, otherwise p < 0.001) (Table 3).

**Table 3:** Patients grouped according to peritoneal transport status [20]

Variable	CAPD	CAPD	CAPD	APD	APD	APD	CCPD	CCPD	CCPD
	Slow	Average	Fast	Slow	Average	Fast	Slow	Average	Fast
Number	13	78	67	74	61	15	34	211	106
Male (%)	6 (46)	38 (49)	39 (58)	32 (43)	37 (61)	11 (73)	34 (32)	121 (57)	74 (70)
Age (years)	54.4 ± 20.1	61.6 ± 15.7	63.7 ± 17.1	55.0 ± 17.2	60.7 ± 13.0	69.5 ± 12.5	50.1 ± 15.8	53.3 ± 16.8	56.3 ± 15.6
Diabetic (%)	5 (39)	32 (41)	30 (45)	19 (26)	20 (33)	5 (33)	34 (32)	78 (37)	45 (43)
Cycles/exchanges	4 (1.5–4)	4 (2–4)	3 (2–4)	6 (5–7)	6 (6–7)	6 (5–7)	7 (6–8)	7 (6–8)	7 (6–8)
Cycle volume (L)	2 (2–2)	2 (2–2)	2 (2–2)	2 (2–2)	2 (2–2)	2 (2–2)	2 (2–2)	2 (2–2)	2 (2–2)
Icodextrin (L/day)	2 (2–2)	2 (2–2)	2 (2–2)	0 (0–0)	0 (0–0)	0 (− 0)	1 (1–2)	1 (1–2)	2 (1–2)
22.7 g/L glucose (L/day)	0 (0–0)	0 (0–0)	0 (0–2)	0 (0–0)	0 (0–0)	0 (0–4)	0 (0–0)	0 (0–5)	0 (0–5)
13.6 g/L glucose (L/day)	3 (− 0 to 6)	4 (0–6)	2 (0–4)	9 (7–10)	9 (6–10)	9 (4–10)	8 (7–10)	10 (9–12)	8 (5–10)
Glucose exposure (mmol/day)	300 (0–450)	319 (0–504)	300 (0–450)	696 (591–755)	705 (600–872)	750 (675–899)	707 (563–945)	839 (675–1082)	816 (686–1005)
Urine (mL/day)	936 (796–1057)	1262 (676–1845)	1103 (320–1616)	1168 (845–1724)	1195 (716–1742)	1198 (872–1416)	975 (438–1500)	964 (412–1572)	700 (384–1338)
Urine sodium (mmol/day)	55 (17–88)	57 (30–102)	46 (18–84)	75 (42–108)	83 (42–115)	71 (46–98)	54 (19–79)	53 (20–100)	40 (14–82)
Weekly (Kt/Vurine)	1.3 (0.8–2.3)	1.3 (0.8–1.8)	1.2 (0.6–1.9)	1.6 (1.0–2.3)	1.3 (0.9–1.9)	1.7 (1.2–2.4)	1.3 (0.4–2.2)	1.1 (0.5–1.8)	0.8 (0.3–1.5)
Weekly (Kt/V_PD_)	1.19 (0.48–1.47)	1.15 (0.67–1.46)	1.05 (0.5–1.55)	1.0 (0.82–1.19)	1.03 (0.65–1.23)	1.07 (0.95–1.23)	1.19 (0.86–1.34)	1.25 (1.04–1.51)	1.4 (1.14–1.72)
Total weekly (Kt/V)	2.52 (2.02–2.84)	2.35 (1.96–3.05)	2.34 (1.74–3.0)	2.65 (2.03–3.32)	2.33 (1.9–3.09)	2.92 (2.07–3.72)	2.41 (1.99–3.2)	2.37 (1.93–3.03)	2.29 (1.81–2.91)
nPNA (g/kg/day)	0.95 (0.82–1.04)	0.94 (0.79–1.09)	0.96 (0.79–1.1)	0.93 (0.76–1.13)	0.98 (0.78–1.14)	0.85 (0.75–0.94)	0.88 (0.74–1.02)	0.85 (0.71–1.01)	0.86 (0.74–1.01)
Peritoneal sodium removal (mmol/day)	50 (22–78)	45 (12–88)	68 (26–132)	24 (20–54)	24 (− 10 to 58)	14.4 (− 3 to 33)	60 (7–90)	50 (6–89)	50 (17–95)
Total sodium removal (mmol/day)	99 ( (63–153)	128 (68–150)	127 (72–183)	115 (79–139)	107 (66–154)	94 ( (62–131)	100 (64–122)	109 (65–162)	110 (63–157)
BMI (kg/m^2^)	25.8 ± 4.1	26.7 ± 4.5	24.3 ± 3.9	26.0 ± 4.0	25.6 ± 4.9	25.5 ± 3.4	27.0 ± 5.7	26.8 ± 4.8	25.3 ± 5.4
MAP (mmHg)	102 ± 16	98 ± 16	101 ± 19	97 ± 15	104 ± 14	94 ± 16	100 ± 14	102 ± 17	104 ± 18
SMMI (kg/m^2^)	9.1 ± 1.2	9.4 ± 3.7	9.4 ± 1.3	9.4 ± 1.6	9.9 ± 1.6	9.7 ± 1.4	9.3 ± 1.8	10.0 ± 2.4	10.1 ± 2.2
FMI (kg/m^2^)	8.8 ± 3.7	9.2 ± 3.7	6.7 ± 3.5	8.7 ± 3.9	7.3 ± 3.9	7.5 ± 2.9	9.8 ± 4.9	8.5 ± 4.0	7.5 ± 4.3
Albumin (g/L)	40 (35–42)	39 (37–41)	35 (31–39)	40 (38–43)	38 (35–40)	35 (32–38)	41 (38–43)	37 (35–40)	36 (32–39)
CRP (mg/L)	4 (2–6)	6 (2–10)	6 (3–16)	3 (1–6)	2 (1–8)	6 (1–12)	3 (2–7)	3 (1–9)	3 (2–6)
Haemoglobin (g/L)	112.8 ± 11.5	114.2 ± 13.9	111.3 ± 13.5	113.9 ± 15.1	113.5 ± 16.1	109.1 ± 19.9	119.7 ± 20.3	110.9 ± 16.5	108.6 ± 15.6

Peritoneal sodium removal adjusted for flush before fill technique. Continuous ambulatory peritoneal dialysis (CAPD) automated peritoneal dialysis (APD) and APD with a day-time exchange (CCPD). Weekly urea clearance (Kt/V), normalised nitrogen appearance rate (nPNA), body mass index (BMI), mean arterial blood pressure (MAP), skeletal muscle mass index (SMMI), fat mass index (FMI) and C reactive protein (CRP). Data expressed ast ingers, percentages, mean ± standard deviation, median (interquartile range)

Univariate analysis demonstrated that net peritoneal sodium loses, adjusted for fill before flush technique were associated with use of peritoneal ultrafiltration volume (r = 0.69, p < 0.001). In addition, there were positive associations with icodextrin and hypertonic glucose exchanges, peritoneal ultrafiltration volume and urea clearance, 4-h dialysate to plasma creatinine ratio, patient age and negatively with 24-h urinary sodium and volume, serum albumin and mean arterial blood pressure (Table 4).

**Table 4:** Univariate Spearman correlation (rho) of variables associated with adjusted peritoneal dialysate sodium removal

Variable	Rho	p
24-h peritoneal ultrafiltration volume	0.69	< 0.001
24-h urine volume	− 0.371	< 0.001
24-h urine sodium	− 0.371	< 0.001
Weekly peritoneal (Kt/Vurea)	0.299	< 0.001
Weekly urinary (Kt/Vurea)	− 0.273	< 0.001
Icodextrin (L/day)	0.251	< 0.001
22.7 g/L glucose (L/day)	0.220	< 0.001
Total weekly (Kt/Vurea)	− 0.111	0.004
4-h dialysate/Plasma_creatinine_	0.108	0.006
Serum albumin	− 0.104	0.007
Age	0.091	0.019
Mean arterial blood pressure	− 0.079	0.041

As the peritoneal sodium balance varied from net sodium retention to losses, a multivariable regression model was analysed comparing greater versus lower sodium losses (Table 4). Daily peritoneal ultrafiltration volume, urea clearance and use of icodextrin and hypertonic glucose exchanges and age remained independently associated with peritoneal sodium losses (Table 5).

**Table 5:** Multivariable step-backward regression model higher than median vs lower than median adjusted peritoneal dialysate sodium

Variable	β	StE-β	St-β	t	95% CL	p
Log UF (mL)	0.23	0.02	0.42	11.9	0.19–0.27	< 0.001
Icodextrin (L/day)	0.14	0.04	0.13	3.7	0.07–0.22	< 0.001
Log Kt/V_PD_	0.31	0.09	0.12	3.2	0.19–0.27	0.001
Age (years)	0.004	0.001	0.11	3.33	0.001–0.01	0.001
22.7 g/L glucose (L/day)	0.01	0.01	0.08	2.1	0.001–0.02	0.034

Non-parametric data was log transformed. Standardised β (St-β), Peritoneal 24-h ultrafiltration (UF), weekly peritoneal Kt/V_urea_ (Kt/V_PD_), Model fit r^2^ 0.28, adjusted r^2^ 0.27. Model checked for collinearity (values all < 1.0) and variance inflation factor (values 1.04–1.18). Standard error (StE), 95% confidence limits (CL)

## Discussion

Besides adequate removal of uraemic toxins, one of the other major treatment goals for PD is regulating volume and sodium balance. Previous reports have suggested a minimum peritoneal ultrafiltration target, once residual renal function has been lost [2]. Prior to the advent of APD cyclers and icodextrin dialysates, then faster peritoneal transporters were reported to at greater risk of PD technique failure [3], thought to be due to failure to achieve adequate ultrafiltration and sodium removal. Although, following the introduction of APD cyclers, reports then suggested no difference in technique survival [6], and several studies have not observed any differences in blood pressure or volume control between different PD treatment modalities [21, 22]. However, a recent meta-analysis reported that treatment with CAPD removed more sodium than APD [23]. As many of these studies were based on the results from a small number of patients, we reviewed peritoneal sodium removal in our cohort of more than 600 patients.

In keeping with some earlier reports, we noted that CAPD patients using an overnight icodextrin exchange had greater daily ultrafiltration volumes and peritoneal sodium losses, compared to those treated by APD with and without a day time icodextrin exchange [11]. Whereas volume in-flow measurements are relatively accurate with APD cyclers, CAPD dialysate bags are over-filled to allow for the flush- before fill technique [17], which has been measured range between 50 and 100 mL [11]. Our patients were taught to allow 15 s for the flush, and when we adjusted for this, then there was no difference in daily ultrafiltration volumes or peritoneal sodium losses compared to patients treated by APD with a day-time icodextrin exchange. Although CAPD losses remained grater than those treated by APD alone, although as

These patients had greater residual renal function and urinary sodium losses, thus over-all losses were similar between modalities, supporting the results of smaller studies [10, 24]. As with many centres we practice incremental dialysis, with peritoneal dialysis prescriptions taking into account residual renal function [25], and as such patients with greater residual renal function and urinary sodium losses had correspondingly lower peritoneal sodium losses.

Compared to some previous studies, peritoneal sodium losses were lower in our study [23]. This may have been due to taking into account the additional sodium load of the flush before fill in CAPD patients, greater residual renal function with greater urinary sodium losses and educating patients to reduce dietary sodium intake.

Sodium is predominantly removed by convection with PD, and as such there was a strong association between ultrafiltration volumes and sodium removal. The association was slightly stronger for CAPD than either APD or APD with a day-time exchange (r^2^ 0.56 vs 0.45 vs 0.44 respectively). It has been suggested that the shorter APD dwell cycles, using glucose dialysates, induce a rapid movement of water through aquaporins, but a slower movement of sodium through co- and active transporters, and as such APD cyclers, so reducing sodium removal when using APD cyclers [11].

Peritoneal sodium removal was also associated with the volume of icodextrin prescribed, supporting other studies which have shown that volume status is better maintained with icodextrin compare to 22.7 g/L glucose exchanges [26]. Whereas some smaller earlier studies did not demonstrate an effect of hypertonic glucose dialysates and peritoneal sodium losses [9], we noted a univariate association, which would be expected as hypertonic dialysates would be expected to increase ultrafiltration [10, 24]. There was a weak association between higher mean arterial blood pressure and lower peritoneal sodium losses, and previous studies have either reported a similar association or no effect of peritoneal sodium losses on blood pressure [9, 27]. In keeping with previous reports, we found no over-all association between transporter status and peritoneal sodium losses [11], which may reflect that the great majority of our patients were prescribed icodextrin, and most patients had residual renal function. However, we noted that both fast-average and fast peritoneal transporters had greater sodium removal with CAPD compare to APD. Faster transporters had shorter APD dwell times compared to slow and slow-average, which may account for the difference in sodium removal [11]. In addition, we also noted an effect of age on peritoneal sodium removal, whether this was due to an increased use of CAPD in older compared to younger patients, or due to changes which occur in the peritoneal membrane with age remains to be determined [28].

As with any observational cross-sectional study we can report associations but not causality. Compared to previous cohorts, we report on over 600 patients predominantly treated with APD cyclers and icodextrin, attending for their first assessment of peritoneal membrane function, and we acknowledge that membrane function may change with time in some patients [29]. Initial investigation showed that CAPD removed more sodium than either APD or APD with a daytime exchange. However, after adjusting for the flush-before fill technique, there was no difference between CAPD and APD with a daytime exchange, although patients treated with APD with a daytime exchange received a greater volume and hypertonic glucose exchanges. Thus, although treatment using APD cyclers can achieve similar peritoneal sodium removal as CAPD, it is at a cost of greater peritoneal exposure to hypertonic exchanges.
